# A machine learning approach to a nine-SNP immunogenetic score for prognostic stratification in cervical cancer

**DOI:** 10.3389/fimmu.2026.1759674

**Published:** 2026-02-06

**Authors:** Sabrina Zidi, Besma Yacoubi-Loueslati, Boutheina Ben Abdelmoumen Mardassi, Wassim Y. Almawi

**Affiliations:** 1 Group of Mycoplasmas, Laboratory of Molecular Microbiology, Vaccinology, and Biotechnology Development, Pasteur Institute of Tunis, University of Tunis El-Manar, Tunis, Tunisia; 2 Laboratory of Mycology, Pathologies, and Biomarkers (LR16ES05), Faculty of Sciences of Tunis, University of Tunis El Manar, Tunis, Tunisia; 3 Faculty of Sciences; University of Tunis El-Manar, Tunis, Tunisia

**Keywords:** cervical cancer, cytokine polymorphisms, IL-10, immunogenetics, machine learning, polygenic risk score, risk stratification, TNF-α

## Abstract

**Background:**

While human papillomavirus (HPV) is the primary driver of cervical cancer (CC), host immune-related genetic variations are thought to influence clinical heterogeneity. The role of combined immune-related single nucleotide polymorphisms (SNPs) in defining patient subgroups remains underexplored in focused, candidate-gene studies.

**Methods:**

We genotyped nine functional SNPs across TNF-α (rs361525, rs1800629), *IL-1β* (rs16944), IFN-γ (rs2430561), *IL-1RN* (rs2234663), *IL-10* (rs3024490, rs1800872, rs1800871), and *IL-6* (rs1474348) in a cohort of 130 Tunisian CC patients. Principal component analysis (PCA), multi-dimensional scaling (MDS), K-means clustering, and random forest modeling were used to explore SNP-based patient subgroups and identify genetic profiles associated with survival.

**Results:**

A high-risk genetic profile, comprising seven SNPs, was identified in 20% of patients. PCA indicated that *IL-10* and TNF-α variants accounted for 38.5% of the observed genetic variance. Unsupervised clustering suggested three distinct SNP-based subgroups with differing genetic architectures. The TNF-α –238 A allele was associated with borderline higher odds of adenocarcinoma (OR 4.57, 95% CI: 0.95–21.95, p=0.050), while the *IL-1β* –511 T allele appeared protective (OR 0.45, 95% CI: 0.19–1.07, p=0.049). Random forest analysis identified the IFN-γ rs2430561 variant as the top predictor of advanced FIGO stage. A nine-SNP polygenic risk score (PRS) was significantly associated with reduced overall survival (HR 2.45, log-rank p<.001) and remained an independent prognostic factor in multivariable analysis. Pathway analysis implicated TNF-α signaling, IL-10 anti-inflammatory, and IL-1 cytokine pathways.

**Conclusions:**

This focused, candidate-gene analysis identifies prognostic SNP-based subgroups and a nine-SNP polygenic risk score associated with survival in cervical cancer. While this work provides a foundation for immunogenetic risk stratification, the findings are derived from a limited SNP panel in a single cohort. Future validation in larger, independent cohorts with genome-wide data is required to confirm these preliminary genetic associations and to determine their relationship to broader molecular subgroups.

## 1 Introduction

Despite the widespread availability of screening methods, increasing accessibility of prophylactic vaccines, and ongoing global awareness campaigns, cervical cancer (CC) incidence persists as a significant global health burden. It remains a leading cause of cancer-related mortality among women worldwide (1–3). This persistent epidemiological trend underscores a critical clinical paradox: while the etiological role of oncogenic human papillomavirus (HPV) subtypes is unequivocally established, a distinct subset of cases presents with no detectable evidence of prior or active HPV infection. This discrepancy necessitates the investigation of complementary oncogenic pathways and cofactors that may drive carcinogenesis independently or synergistically.

Mounting evidence suggests that host genetic predispositions constitute primary cofactors in CC development (4). Population-based registry studies provide compelling evidence for a significantly elevated familial relative risk (FRR), indicating a pronounced tendency for the disease to aggregate within families (5–7). Familial aggregation studies suggest that the inherited tendency to CC is primarily polygenic, driven by multiple common, low-penetrance variants interacting with environmental and viral cofactors, analogous to the documented FRR for breast cancer (5–7). Nevertheless, a distinct contrast emerges; unlike breast and ovarian cancers, large, multiplex pedigrees are seldom reported (4, 6, 8–10). This paucity of high-density familial cases suggests that highly penetrant germline mutations are infrequent and that the heritable liability is likely polygenic. This model proposes that susceptibility is mediated by a combination of common, low-penetrance genetic variants that collectively moderate risk, potentially through interactions with environmental and viral oncogenic factors.

Guided by this rationale, research into genetic susceptibility has extensively employed a candidate-gene approach, focusing on genes implicated in immune regulation, tumor suppression, and DNA damage response. Reported associations include variants in genes such as *TP53* (11–13), *MDM2* (12, 14, 15), *ATM* (16), *BRIP1* (17), *CDKN1A* (18–20), *CDKN2A* (21), *FACNA*, *FANCC*, *FANCL* (22), *XRCC1* (23–25), and *XRCC3* (26). Concurrently, significant focus has been directed towards immune response genes, including those encoding T-cell surface molecules *CD83* (27, 28) and *CTLA4* (29), inflammasome components *CARD8* (30), and cytokines; *TNFA* (29–32), interleukins (33–36), *TGFB1* (37) and *IFNG* (18, 38).

Notwithstanding these considerable efforts, the candidate-gene era has been challenged by a pervasive lack of reproducibility. Most proposed risk variants, including the extensively debated *TP53* Arg72Pro polymorphism (39), have failed to be consistently replicated or to attain genome-wide statistical significance in large-scale studies, with the notable exception of certain *HLA* alleles (40). Technological advancements have primarily addressed this limitation over the past decade. The advent of genome-wide association studies (GWAS), enabling the agnostic interrogation of millions of variants across the genome, has provided more robust evidence for additional risk loci.

Large-scale GWAS and post-GWAS analyses demonstrate that cervical cancer susceptibility is highly polygenic, with common SNPs accounting for substantial heritability (40). Mendelian randomization and immune-cell GWAS implicate inherited variations in cytokine signaling and T-cell activation as causal risk determinants (38, 41). These findings suggest that host immune architecture, rather than isolated loci, governs HPV persistence and malignant progression. Replicated risk factors include polymorphisms in TNF-α, IL-1β, IL-6, and IL-10, with ethnicity and HPV status influencing effect sizes (13, 29, 31, 34). While cumulative risk models outperform single-SNP predictors [ (37), current polygenic risk scores (PRS) often lack functional relevance (40). Consequently, biologically grounded PRS frameworks focusing on population-specific cytokine dysregulation are required to improve risk stratification.

Building upon this foundational work, the present study was initiated. We used data from a GWAS conducted by our research team within a representative Tunisian cohort to select single-nucleotide polymorphisms (SNPs) that showed significant associations with CC susceptibility. The primary objectives of this follow-up investigation are to define a population-specific immunogenetic predisposition profile for CC in Tunisian women and to correlate identified genetic risk factors with distinct epidemiological and clinical characteristics. This study aims to complement GWAS discovery by evaluating a biologically grounded, cytokine−focused polygenic risk model in North African Tunisian population, integrating functional immune regulatory variants with machine−learning patient stratification and survival analysis.

## 2 Patients and methods

### 2.1 Study subjects

The study population, recruitment criteria, and sample collection procedures were described previously (42). Briefly, it included 130 women with histologically confirmed CC recruited from Salah Azeiz Oncology Institute (SAI, Tunis, Tunisia). The cancer diagnosis was established by clinical examination and biopsy findings, and two senior SAI pathologists confirmed it. Clinical data were collected through self-reported questionnaires, case record reviews, and personal interviews. Tumor staging was according to the International Federation of Gynecology and Obstetrics (FIGO) classification (www.figo.org). Peripheral blood EDTA-anticoagulated specimens were collected from CC patients before radiation therapy or chemotherapy. Genomic DNA was extracted using QIAamp_ DNA Blood Mini Kit, according to the manufacturer’s instructions (Qiagen GmbH, Hilden, Germany). Study subjects were from different zones of Tunisia, and were asked to sign a consent form agreeing to participate in the study; all institutional ethics requirements were met.

### 2.2 Genotyping

The selection of the studied SNPs was guided by evidence from previous case–control and genome-wide association studies reporting significant associations with CC risk (42–46). Accordingly, only variants previously identified as positively associated with CC susceptibility were retained for this analysis. Based on this rationale, a targeted genotyping approach was applied to nine candidate SNPs located in key genes implicated in inflammation and immune regulation within a cohort of 130 patients: rs361525 and rs1800629 (*TNF-α*), rs16944 (*IL-1β*), rs2430561 (*IFN-γ*), rs2234663 (*IL-4R*), rs3024490, rs1800872, and rs1800871 (*IL-10*), and rs1474348 (*IL-10R*). Genotyping was performed by the allelic (VIC and FAM-labelled) discrimination method. Assay-on-demand TaqMan assays were ordered from Applied Biosystems (Foster City, NJ). The reaction was performed in 6 μl volumes on StepOne/StepOne Plus real-time PCR systems, as recommended by the manufacturer (Applied Biosystems). Quality control measures to assess reproducibility of the genotyping procedure included: 1) replicate genotyping of 10% of blinded samples showing >99% concordance; 2) inclusion of negative controls in each run; 3) independent assessment of ambiguous genotype calls by two investigators; and 4) exclusion of samples with >20% missing genotype data.

### 2.3 Statistical analysis

Statistical analyses were performed using IBM SPSS Statistics (Version 31; IBM Corp., Armonk, NY, USA) and RStudio (Posit Software, Boston, MA, USA; R version 4.x, latest release). Descriptive statistics were expressed as percentages for categorical variables. Inter-group significance was assessed using the Pearson χ² test. Logistic regression was used to estimate odds ratios (ORs) and 95% confidence intervals (95% CIs). All analyses were performed under the assumption of an additive genetic effect model. Genotype distributions for all SNPs were tested for deviation from Hardy-Weinberg equilibrium (HWE) using chi-square goodness-of-fit tests. Given the exploratory nature of this candidate gene study involving 9 SNPs and multiple clinical comparisons, we report uncorrected p-values and acknowledge that the findings should be interpreted cautiously pending replication. Genotypes with call rates <95% were excluded. The remaining missing data were assumed to be missing at random, not imputed, and were omitted from locus-specific analyses but retained for others.

We applied Benjamini–Hochberg FDR correction to all primary SNP–phenotype association tests, including allelic and genotypic comparisons across FIGO stage, histology, and epidemiological strata, to control type I error. Adjusted q−values are reported with nominal p−values. Machine−learning analyses (PCA, clustering, random forest) were treated as exploratory and not corrected for multiplicity. The polygenic risk score (PRS) was calculated as an unweighted allelic burden by summing risk alleles across 9 SNPs, an approach chosen to avoid instability and overfitting in this modest cohort. Random Forest modeling was used as a multivariate, non-linear classification tool to rank SNPs according to their contribution to prediction accuracy. Variable-importance scores represent relative predictive influence within the model and do not imply biological causality or mechanistic dominance.

In RStudio, a correlation matrix was computed to explore pairwise associations between single-nucleotide polymorphisms (SNPs). Dimensionality reduction approaches, including Principal Component Analysis (PCA) with variable plots and Multidimensional Scaling (MDS), were applied to visualize patterns of genetic variation. Heatmaps were generated to display SNP distributions, and unsupervised K-means clustering (k = 3) was performed on PCA outputs to classify cases into genetic clusters. Random Forest modelling was employed to predict clinical outcomes and identify the most informative SNP predictors. Additionally, functional pathway enrichment analysis (enrichR) was performed to identify biological functions associated with significantly correlated SNPs. *Post-hoc* power calculations were performed to assess the study’s ability to detect associations of specified effect sizes at α=0.05. A Venn diagram was generated using stringdb (https://string-db.org/), and a protein–protein interaction network was obtained using webtools (https://jvenn.toulouse.inrae.fr/app/index.html).

## 3 Results

### 3.1 Study design

Baseline and demographic characteristics of the study population are described in **Table 1**. Of the 130 cases examined, the cohort had a mean age of 52.3 ± 7.6 years, with the majority (53.9%) of patients aged 51–60 years. Most participants were married or in a stable relationship (85.4%) and reported having a single sexual partner (86.2%). Over half of the cohort (52.3%) was peri-menopausal, and the vast majority (80.7%) were not using hormonal contraception. A minority (20.7%) were identified as tobacco users. Clinically, a family history of cancer was reported in 63% of cases. The predominant histology was squamous cell carcinoma (73.8%), followed by adenocarcinoma (18.5%) and sarcoma (7.7%). More than half of the cases (59.2%) were diagnosed at an early FIGO stage. According to the TNM classification, the tumors were primarily distributed across T1 (29.2%), T2 (30%), and T3 (26.9%) stages, with most patients presenting with no nodal involvement (N0, 74.6%) and no distant metastasis (M0, 87.7%).

**Table 1:** Patient basal characteristics (n = 130).

Characteristics	Cases (n=130)
*Environnemental characteristics*	
Age (mean ± SD)	52.3±7.6
30–40 yr	13 (10%)
41–50 yr	29 (22.3%)
51–60 yr	70 (53.9%)
61–70 yr	18 (13.8%)
Marital status	
+/-^1^	111(85.4%)/19(14.6%)
Sexual partner	
0	2 (1.5%)
1	112 (86.2%)
> 2	16 (12.3%)
Menopause status	
Pre-menopausal Peri-menopausal	28 (21.5%) 68 (52.3%)
Post-menopausal	34 (26.2%)
Hormonal contraception	
+/-^2^	105(80.7%)/25(19.3%)
Tobacco users	
+/-^3^	27(20.7%)/103(79.3%)
*Clinical characteristics*	
Family history of cancer	
+/-^4^	82(63%)/ 48(37%)
Histology	
Squamous cell carcinoma	96 (73.8%)
Adenocarcinoma	24 (18.5%)
Sarcoma	10 (7.7%)
FIGIO staging	
Early stages	77 (59.2%)
Late stages	43 (40.8%)
TNM classification	
T staging	
T1	38 (29.2%)
T2	39 (30%)
T3	35 (26.9%)
T4	18 (13.9%)
N staging	
N0	97 (74.6%)
N1	33 (25.4%)
M staging	
M0	114 (87.7%)
M1	16 (12.3%)

+/−^1^: (+) Married, (−) Single; +/−^2^: (+) Yes, (−) No; +/−^3^: (+) Yes, (−) No, +/−^4^: (+) presence of family cancer, (-) absence of family cancer, FIGO International Federation of Gynecology and Obstetrics; n: Number of women.

### 3.2 Immunogenetic profiling of cervical cancer

**Figure 1:**
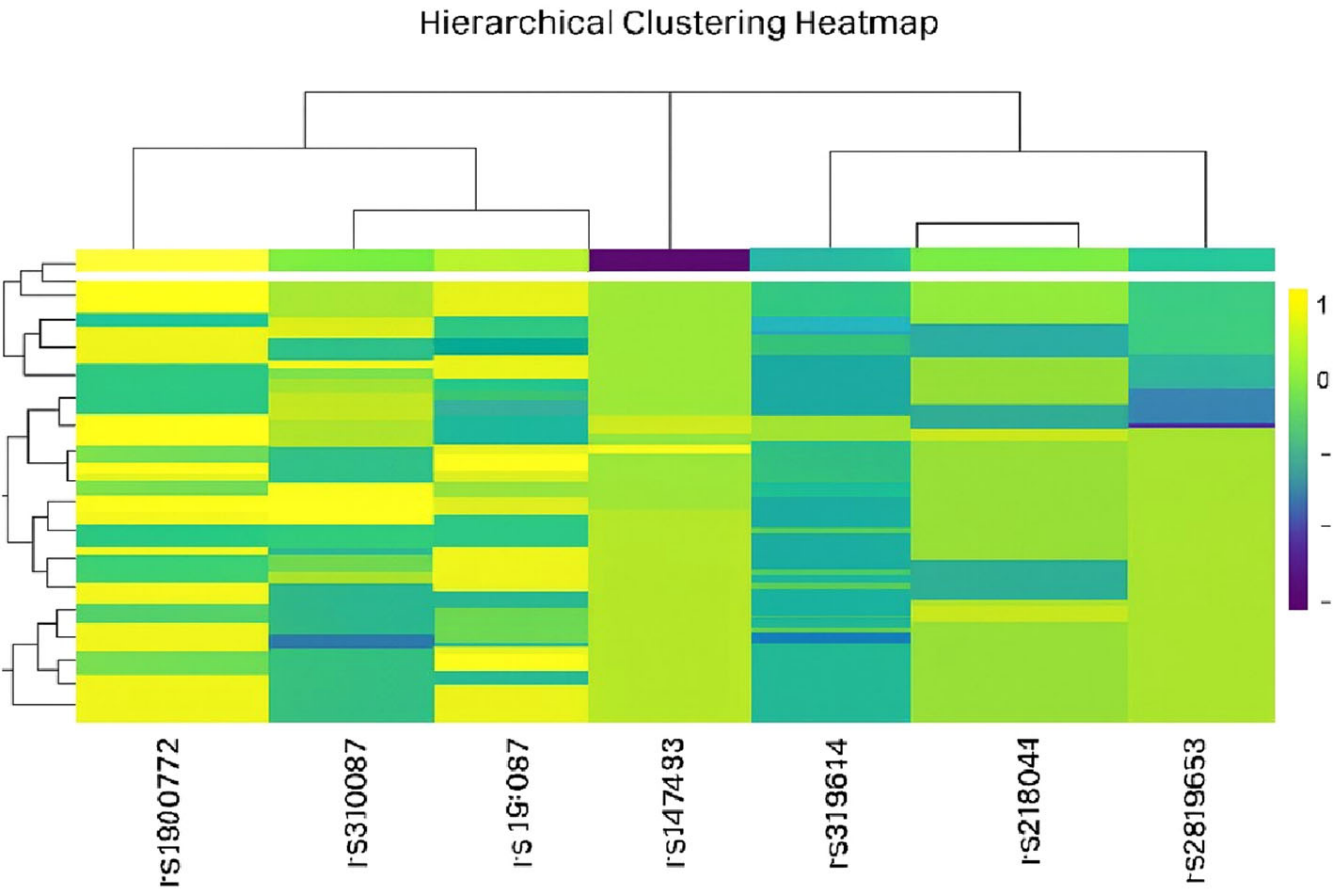
Genotype heatmap displaying the distribution of nine immunogenetic SNP variants across cervical cancer patients. The heatmap displays genotype patterns across patients (columns) and selected SNPs (rows) for patients with ≥2 heterozygous SNP variants (n=117 out of N = 130). Columns are labeled with individual patient identifiers. Rows correspond to the following SNPs: rs1900772 rs310087, rs19:067, rs147493, rs319614, rs218044, and rs2819653. Hierarchical clustering of both rows and columns reveals patterns of genotype similarity among patients and allele co-segregation among SNPs. Notable linkage disequilibrium and coordinated inheritance are observed between IL-10 promoter variants (rs1800872 and rs1800871), supporting their co-inheritance within inflammatory pathways. **Color key:** • **Purple:** Homozygous variant • **Blue/Teal:** Heterozygous • **Green/Yellow:** Wild-type.

Comprehensive immunogenetic analysis of our CC cohort revealed a substantially high allelic burden across the nine studied promoter polymorphisms. Our data indicate widespread prevalence of genetic variants, with a significant majority of patients (71.5%) harboring variation in at least 4 polymorphisms. Hierarchical heatmap analysis (**Figure 1**), performed on all study patients who met the inclusion criterion of having ≥2 heterozygous SNP variants (n = 117 of 130), revealed a discrete high-burden subgroup characterized by the presence of seven risk alleles:; A for rs1800629 (*TNF-α*), C for rs16944 (*IL-1β*), A for rs2430561 (*IFN-γ*), L for rs2234663 (*IL-1RN*), T for rs3024490, A for rs1800872 and T for rs1800871 (*IL-10*), defining a polygenic susceptibility cluster (**Figure 1**).

Hierarchical clustering analysis of SNP variation revealed distinct patient subgroups based on their genetic profiles (**Figure 1**). The dendrogram indicated optimal separation into three major clusters: Cluster 1 comprising 4 patients (3.4%), Cluster 2 comprising 24 patients (20.5%), and Cluster 3 comprising 89 patients (76.1%). These clusters exhibited distinct patterns of SNP variation across the nine analyzed loci (rs1000272, rs1000971, rs1800629, rs147348, rs16944, rs3024400, rs2400591, rs361625, and rs2234663). The heatmap visualization demonstrated that Cluster 1 represents a genetically distinct subgroup with unique variation patterns, Cluster 2 displays intermediate genetic diversity, and Cluster 3 includes the majority of patients with more homogeneous SNP profiles. The hierarchical structure further revealed that Clusters 2 and 3 are more closely related to each other than to Cluster 1, suggesting the presence of two major genetic lineages within the study population. The clustering patterns appeared robust, as evidenced by clear separation between clusters and consistent grouping in the dendrogram.

The resulting hierarchical structure delineated clear patterns of genetic variation and suggested potential linkage disequilibrium between specific loci. The heatmap corroborated the statistical findings by demonstrating limited heterogeneity in certain SNPs, such as rs1800872 and rs1474348, which exhibited largely uniform genotypic distributions. In contrast, loci including rs361525 and rs1800629 showed pronounced variability, with clear segregation of patients into heterozygous and homozygous genotypes. Furthermore, the visualization confirmed the presence of rare alleles, such as the *L/*2 variant of rs2234663, which was restricted to a specific subset of patients. Notably, visual inspection suggested cosegregation of alleles across seven high-risk SNPs, suggesting a potential haplotype block defining the identified high-burden subgroup.

Collectively, these findings indicate a stepwise increase in genetic risk, ranging from a baseline associated with≥4 polymorphic variants to a high-risk threshold defined by the presence of 7 specific polymorphisms. This pattern suggests a potential dose-dependent effect of a pro-inflammatory genetic background on disease susceptibility. The polymorphic signature identified in this study, particularly the high-burden profile, may represent a valuable predictive biomarker for stratifying individuals at increased risk of developing CC.

### 3.3 Allelic and genotypic frequency according to clinical feature in study cases

We analyzed SNP genotype distributions across FIGO stages to explore potential links between candidate immune variants and cervical cancer progression. Initial statistical analysis did not reveal significant associations for cytokine polymorphisms with disease stage, despite visual variability in genotypic patterns (**Table 2**). Upon exploratory stratification by histological subtype, several suggestive associations emerged. The *TNF-α* -238A allele demonstrated a borderline, non-significant association with adenocarcinoma risk (OR 4.57, 95% CI 0.95–21.95, p = 0.050), an observation that requires caution due to the limited subgroup sample size. Notably, the A/A genotype was absent in squamous cell carcinoma cases. Conversely, a trend suggestive of a protective effect was observed for the *IL-1β* -511T allele, with the T/T genotype showing borderline association with reduced adenocarcinoma risk and the combined C/T+T/T group associated with lower invasive carcinoma risk (OR: 0.45, p = .049). These patterns suggest the hypothesis that *TNF-α* and *IL-1β* variants may play opposing roles in cervical carcinogenesis in a subtype-specific manner. After FDR correction, none of the histology-stratified associations remained statistically significant at q < 0.05, indicating that these findings should be interpreted as hypothesis-generating rather than confirmatory.

**Table 2:** **A**llelic and genotypic frequency according clinical feature in study cases.

Genes/rs	Genotypes	Carcinoma n Situ N (%)	Adenocarcinoma N (%)	Squamous cell carcinoma N (%)	*p_value_*	OR (95% CI)	Stage I N (%)	Stage II N (%)	Stage III N (%)	Stage IV N (%)	P*_value_	OR (95% CI)
***TNFα-238 rs361525***	G/G	79 (60.76)	14 (10.76)	9 (6.92)	**Reference**		30 (23.07)	31 (23.84)	27 (20.76)	14 (10.76)	**Reference**	
	G/A	14 (10.76)	6 (4.61)	1 (0.76)	0.217	1.71(0.61-4.75)	6 (4.61)	6 (4.61)	6 (4.61)	3 (2.30)	0.582	1.07(0.36-2.94)
	A/A	3 (2.30)	4 (3.07)	0 (0.00)	**0.050**	**4.57(0.95-21.95)**	2 (1.53)	2 (1.53)	2 (1.53)	1 (0.76)	0.664	1.04(0.19-5.66)
	(G/A+A/A)	17 (13.07)	10 (7.69)	1 (0.76)	**0.049**	**2.22(0.91-5.40)**	8 (6.15)	8 (6.15)	8 (6.15)	4 (3.07)	0.565	1.04(0.41-2.62)
***TNFα-308* rs1800629**	A/A	13 (10.0)	3 (2.30)	2 (1.53)	**Reference**		5 (3.84)	6 (4.61)	4 (3.07)	3 (2.30)	**Reference**	
	A/G	51 (39.23)	12 (9.23)	8 (6.15)	0.611	1.01(0.32-3.23)	24 (18.46)	20 (15.38)	18 (13.84)	9 (6.92)	0.426	0.75(0.24-2.36)
	G/G	32 (24.61)	9 (6.92)	0 (0.00)	0.430	0.73(0.20-2.60)	9 (6.92)	13 (10.00)	13 (10.00)	6 (4.61)	0.430	1.36(0.38-4.86)
	(A/G+G/G)	83 (63.84)	21 (16.15)	8 (6.15)	0.530	0.9(0.29-2.76)	33 (25.38)	33 (25.38)	31 (23.84)	15 (11.53)	0.563	0.92(0.30-2.78)
***IL 1β- 511*** **rs16944**	C/C	19 (14.61)	7 (5.38)	5 (3.84)	**Reference**		10 (7.69)	5 (3.84)	11 (8.46)	5 (3.84)		**Reference**
	C/T	57 (43.84)	13 (10.0)	5 (3.84)	0.099	0.5(0.20-1.22)	22 (16.92)	28 (21.53)	16 (12.30)	9 (6.92)	0.468	1.14(0.46-2.82)
	T/T	20 (15.38)	4 (3.07)	0 (0.00)	**0.051**	**0.31(0.08-1.15)**	6 (4.61)	6 (4.61)	8 (6.15)	4 (3.07)	0.388	1.42(0.43-4.70)
	(C/T+TT)	77 (59.23)	17 (13.07)	5 (3.84)	**0.049**	**0.45(0.19-1.07)**	28 (21.53)	34 (26.15)	24 (18.46)	13 (10.00)	0.415	1.2(0.50-2.88)
***IFN-ϒ873* rs2430561**	A/A	18 (13.84)	4 (3.07)	3 (2.30)	**Reference**		5 (3.84)	7 (5.38)	8 (6.15)	5 (3.84)	**Reference**	
	A/T	43 (33.07)	10 (7.69)	4 (3.07)	0.471	0.83(0.28-2.41)	21 (16.15)	13 (10.0)	16 (12.30)	7 (5.38)	0.103	0.42(0.14-1.31)
	T/T	35 (26.92)	10 (7.69)	3 (2.30)	0.571	0.95(0.32-2.81)	12 (9.23)	19 (14.61)	11 (8.46)	6 (4.61)	0.432	0.75(0.23-2.43)
	(A/T+T/T)	78 (60.0)	20 (15.38)	7 (5.38)	0.497	0.8(0.33-2.36)	33 (25.38)	32 (24.61)	27 (20.76)	13 (10.00)	0.189	0.54(0.18-1.57)
***IL1RA* rs2234663**	L/L	65 (50.0)	18 (13.84)	7 (5.38)	**Reference**		27 (20.76)	24 (18.46)	25 (19.23)	14 (10.76)	**Reference**	
	L/2*	20 (15.38)	3 (2.30)	1 (0.76)	0.200	0.52(0.16-1.67)	9 (6.92)	8 (6.15)	3 (2.30)	4 (3.07)	0.319	0.71(0.27-1.83)
	2*/2*	11 (8.46)	2 (1.53)	2 (1.53)	0.600	0.94(0.27-3.24)	2 (1.53)	7 (5.38)	7 (5.38)	0 (0.00)	0.123	3.1(0.63-14.11)
	(L/2*+2*/2*)	31 (23.84)	5 (3.84)	3 (2.30)	0.261	0.67(0.27-1.65)	11 (8.46)	15 (11.53)	10 (7.69)	4 (3.07)	0.472	1.12(0.49-2.58)
***IL10* rs3024490**	G/G	44 (33.84)	10 (7.69)	5 (3.84)	**Reference**		16 (12.30)	15 (11.53)	21 (16.15)	7 (5.38)	**Reference**	
	G/T	4 (3.07)	0 (0.00)	0 (0.00)	0.326	Undefined	0 (0.00)	2 (1.53)	0 (0.00)	2 (1.53)	0.299	Undefined
	T/T	48 (36.92)	14 (10.76)	5 (3.84)	0.433	1.16(0.52-2.56)	22 (16.92)	22 (16.92)	14 (10.76)	9 (6.92)	0.308	0.76(0.35-1.64)
	(G/T+TT)	52 (40.00)	14 (10.76)	5 (3.84)	0.512	1.07(0.48-2.35)	22 (16.92)	24 (18.46)	14 (10.76)	11 (8.46)	0.387	0.82(0.38-1.77)
***IL10* rs1800872**	A/A	6 (4.61)	0 (0.00)	0 (0.00)	**Reference**		1 (0.76)	3 (2.30)	0 (0.00)	2 (1.53)	**Reference**	
	A/C	47 (36.15)	14 (10.76)	5 (3.84)	0.146	Undefined	20 (15.38)	22 (16.92)	16 (12.30)	8 (6.15)	0.814	0.46(0.05-4.19)
	C/C	43 (33.07)	10 (7.69)	5 (3.84)	0.186	Undefined	17 (13.07)	14 (10.76)	19 (14.61)	8 (6.15)	0.877	0.49(0.05-4.54)
	(A/C+C/C)	90 (96.23)	24 (18.46)	10 (7.69)	0.155	Undefined	37 (28.46)	36 (27.69)	35 (26.92)	16 (12.30)	0.815	0.47(0.05-4.16)
***IL10* rs1800871**	C/C	40 (30.76)	8 (6.15)	3 (2.30)	**Reference**		15 (11.53)	15 (11.53)	17 (13.07)	4 (3.07)	**Reference**	
	C/T	52 (40.00)	15 (11.53)	7 (5.38)	0.442	0.83(0.33-2.09)	22 (16.92)	23 (17.69)	18 (13.84)	11 (8.46)	0.565	0.98(0.45-2.15)
	T/T	4 (3.07)	1 (0.76)	0 (0.00)	0.710	0.90(0.09-8.98)	1 (0.76)	1 (0.76)	0 (0.00)	3 (2.30)	1	1.66(0.17-16.17)
	(C/T+C/C)	56 (43.07)	16 (12.30)	7 (5.38)	0.227	1.49(0.65-3.40)	23 (17.96)	24 (18.46)	18 (13.84)	14 (10.76)	0.561	1.01(0.46-2.19)
***IL6* rs1474348**	G/G	5 (3.84)	1 (0.76)	1 (0.76)	**Reference**		2 (1.53)	0 (0.00)	3 (2.30)	2 (1.53)	**Reference**	
	G/C	24 (18.46)	3 (2.30)	3 (2.30)	0.479	0.62(0.09-4.04)	10 (7.69)	9 (6.92)	7 (5.38)	4 (3.07)	1	0.80(0.13-4.87)
	C/C	67 (51.53)	20 (15.38)	6 (4.61)	0.634	0.97(0.17-5.31)	26 (20.00)	30 (23.07)	25 (19.23)	12 (9.23)	1	1.03(0.18-5.64)
	(G/C+CC)	91 (70.00)	23 (17.96)	9 (6.92)	0.588	0.87(0.16-4.75)	36 (27.69)	39 (30.00)	32 (24.61)	16 (12.30)	1	0.96(0.17-5.21)

*P^1^_value_*: Carcinoma In Situ vs Squamous Cell Carcinoma+ Adenocarcinoma *; P^2^_value_*: Stage 1 vs Stages 2+3+4; Study subjects comprised 130 CC cases; N: Number of subjects (percent total within group).

Values in bold are statistically significant at the 5 % level.

### 3.4 Allelic and genotypic frequency according to epidemiological characteristics

Analysis of epidemiological characteristics reveals significant gene-environment interactions. *IL-1RA* VNTR 2* allele showed elevated risk in women with familial cancer history (OR: 3.26, p <.001), tobacco users (OR: 1.95, p = .028), and a trend in postmenopausal women was also noted (OR: 1.80, p = .050) (**Table 3**). Conversely, *the TNF-α* -308G allele was protective against familial cancer (OR: 0.56, p = .018), while *IL-10* rs3024490 showed mixed associations: protective in familial cancer (OR: 0.58, p = .027) but susceptible in smokers (OR: 1.87, p = .029). The *IL-6* C allele (rs1474348) was also associated with familial cancer risk (OR: 1.91, p = .037). Contraceptive use showed minimal genetic associations, and marital status had no significant impact. Collectively, this suggests that cytokine gene variants influence CC risk by interacting with hormonal status, genetic predisposition, and environmental exposures, rather than acting as independent risk factors. While the association between *IL-1RA* VNTR and family history of cancer remained statistically significant following FDR adjustment (q < 0.05), the smoking- and menopause-related associations did not survive multiple-testing correction.

**Table 3:** Allelic and genotypic frequency according epidemiological characteristics in study cases.

SNP	Genotypes & Alleles	Study cases (n=130)					*P^1^_value_*	OR (95% CI)^2^	*P^2^_value_*	OR (95% CI)^2^	*P^3^_value_*	OR (95% CI)^3^	*P^4^_value_*	OR (95% CI)^4^	*P^5^_value_*	OR (95% CI)^5^
		Marital status	Menopausal status	Familial cancer	Tobacco users	Contraception use										
**TNFα-238** **rs361525**	G/G	7/0	5/2	3/4	0/7	5/2	–	***Reference***	**-**	***Reference***	**-**	***Reference***	**-**	***Reference***	**-**	***Reference***
	G/A	18/3	12/9	11/10	5/16	19/2	0.724	2.47[1.03-5.92]	0.569	1.15[0.76-1.74]	0.668	0.68[0.12-3.82]	0.392	1.1[0.85-1.42]	0.193	0.26[0.05-1.24]
	A/A	94/8	79/23	68/34	22/80	81/21	0.443	2.39[0.99-5.77]	0.738	1.09[0.72-1.65]	0.203	0.37[0.07-1.77]	0.513	1.09[0.84-1.42]	0.441	0.46[0.12-1.72]
	(G/A+A/A)	112/11	91/32	.79/44	27/96	100/23	0.897	2.41[1.00-5.83]	0.704	1.10[0.73-1.67]	0.256	0.41[0.08-1.95]	0.515	1.09[0.84-1.41]	0.376	0.42[0.11-1.59]
	**G**	32/3	22/13	17/18	5/30	29/6	–	***Reference***	**-**	***Reference***	**-**	***Reference***	**-**	***Reference***	**-**	***Reference***
	**A**	206/19	170/55	147/78	49/176	181/44	0.980	1.78[0.84-3.76]	0.146	0.54[0.25-1.15]	**0.043**	**0.5[0.24-1.02]**	0.217	0.59[0.22-1.62]	0.471	1.17[0.45-3]
**TNFα-308** **rs1800629**	A/A	18/0	11/7	5/13	2/16	15/3	–	***Reference***	**-**	***Reference***	**-**	***Reference***	**-**	***Reference***	**-**	***Reference***
	A/G	63/8	52/19	49/22	16/55	56/15	0.137	2.08[1.0-4.32]	0.385	0.57[0.19-1.69]	**0.001**	**0.17[0.05-0.54]**	0.762	1.04[0.81-1.34]	0.963	1.05[0.51-2.14]
	G/G	38/3	33/8	28/13	9/32	34/7	0.242	1.95[0.93-4.07]	0.192	0.38[0.11-1.29]	**0.004**	**0.17[0.05-0.60]**	0.733	1.05[0.82-1.34]	0.916	0.96[0.46-2]
	(A/G+G/G)	101/11	85/27	77/35	25/87	90/22	0.350	1.7[0.96-3.01]	0.246	0.49[0.17-1.41]	**0.001**	**0.17[0.05-0.52]**	0.771	1.04[0.81-1.33]	0.912	1.02[0.5-2]
	**A**	99/8	74/33	59/48	20/87	86/21	–	***Reference***	**-**	***Reference***	**-**	***Reference***	**-**	***Reference***	**-**	***Reference***
	**G**	139/14	118/35	105/48	34/119	124/29	0.821	1.24[0.50-3.08]	0.155	0.66[0.38-1.16]	**0.018**	**0.56[0.33-0.93]**	0.297	0.80[0.43-1.49]	0.507	0.95[0.51-1.78]
**IL 1β- 511** **rs16944**	C/C	28/3	24/7	20/11	4/27	21/10	–	***Reference***	**-**	***Reference***	**-**	***Reference***	**-**	***Reference***	**-**	***Reference***
	C/T	70/5	52/23	43/32	17/58	64/11	0.142	1.52[0.90-2.59]	0.481	1.51[0.57-4.01]	0.321	1.35[0.56-3.21]	0.812	1.03[0.81-1.32]	**0.038**	**0.36[0.13-0.96]**
	T/T	21/3	20/4	19/5	6/18	20/4	0.079	1.66[0.97-2.84]	0.806	0.68[0.17-2.68]	0.188	0.47[0.13-1.63]	0.761	1.04[0.81-1.34]	0.660	0.82[0.45-1.51]
	(C/T+TT)	91/8	72/27	62/37	23/76	84/15	0.126	1.54[0.91-2.61]	0.815	1.28[0.49-3.32]	0.513	1.08[0.46-2.51]	0.842	1.03[0.80-1.31]	**0.035**	**0.37[0.14-0.95]**
	**C**	126/11	100/37	83/54	25/112	106/31	**-**	***Reference***	**-**	***Reference***	**-**	***Reference***	**-**	***Reference***	**-**	***Reference***
	**T**	112/11	92/31	81/42	29/94	104/19	0.826	1.12[0.46-2.69]	0.778	0.91[0.52-1.58]	0.226	0.79[0.48-1.32]	0.182	0.72[0.39-1.31]	0.094	0.62[0.33-1.17]
**IFN-ϒ873 rs2430561**	A/A	23/2	18/7	14/11	4/21	22/3	**-**	***Reference***	**-**	***Reference***	**-**	***Reference***	**-**	***Reference***	**-**	***Reference***
	A/T	51/6	43/14	39/18	12/45	44/13	0.134	1.42[0.92-2.18]	0.786	0.83[0.28-2.41]	0.201	0.58[0.22-1.54]	0.690	1.05[0.82-1.35]	0.629	1.22[0.65-2.29]
	T/T	45/3	35/13	29/19	11/37	39/9	0.175	1.37[0.89-2.13]	0.740	0.95[0.32-2.81]	0.453	0.83[0.31-2.21]	0.714	1.05[0.82-1.34	0.800	1.14[0.59-2.33]
	(A/T+T/T)	96/9	78/27	68/37	23/82	93/22	0.154	1.39[0.90-2.15]	0.780	0.89[0.33-2.36]	0.276	0.69[0.28-1.67]	0.721	1.05[0.82-1.34	0.725	1.17[0.63-2.17]
	**A**	97/10	79/28	67/40	20/87	88/19	**-**	***Reference***	**-**	***Reference***	**-**	***Reference***	**-**	***Reference***	**-**	***Reference***
	**T**	141/12	113/40	97/56	34/119	122/31	0.659	0.82[0.34-1.98]	0.553	0.99[0.56-1.75]	0.499	0.96[0.57-1.61]	0.297	0.80[0.43-1.49]	0.367	1.17[0.62-2.21]
**IL1RA** **rs2234663**	L/L	81/9	71/19	61/29	18/72	72/18	–	***Reference***	**-**	***Reference***	**-**	***Reference***	**-**	***Reference***	**-**	***Reference***
	L/2*	24/0	14/10	10/14	4/20	20/4	0.234	1.02[0.68-1.55]	**0.039**	**2.66[1.02-6.94]**	**0.018**	**2.94[1.16-7.42]**	0.775	1.04[0.82-1.3]	0.683	1.11[0.74-1.65]
	2*/2*	14/2	11/5	11/5	5/11	13/3	0.627	1.13[0.75-1.70]	0.275	1.69[0.52-5.48]	0.593	0.95[0.30-3.00]	0.242	0.55[0.16-1.78]	0.591	1.14[0.75-1.74]
	(L/2*+2*/2*)	38/2	25/15	21/19	9/31	33/7	0.545	1.04[0.70-1.57]	**0.042**	**2.24[0.99-5.07]**	0.071	1.9[0.88-4.07]	0.457	0.86[0.34-2.12]	0.470	0.84[0.32-2.22]
	**L**	186/18	156/48	132/72	40/164	164/40	**-**	***Reference***	**-**	***Reference***	**-**	***Reference***	**-**	***Reference***	**-**	***Reference***
	2*	52/4	36/20	32/57	14/112	46/10	0.897	1.07[0.72-1.59]	**0.050**	**1.80[0.95-3.41]**	**0.000**	**3.26[1.94-5.49]**	**0.028**	**1.95[1.01-3.75]**	0.467	0.89[0.41-1.91]

Values in bold are statistically significant at the 5 % level.

### 3.5 Correlation between studied SNPs

**Figure 2:**
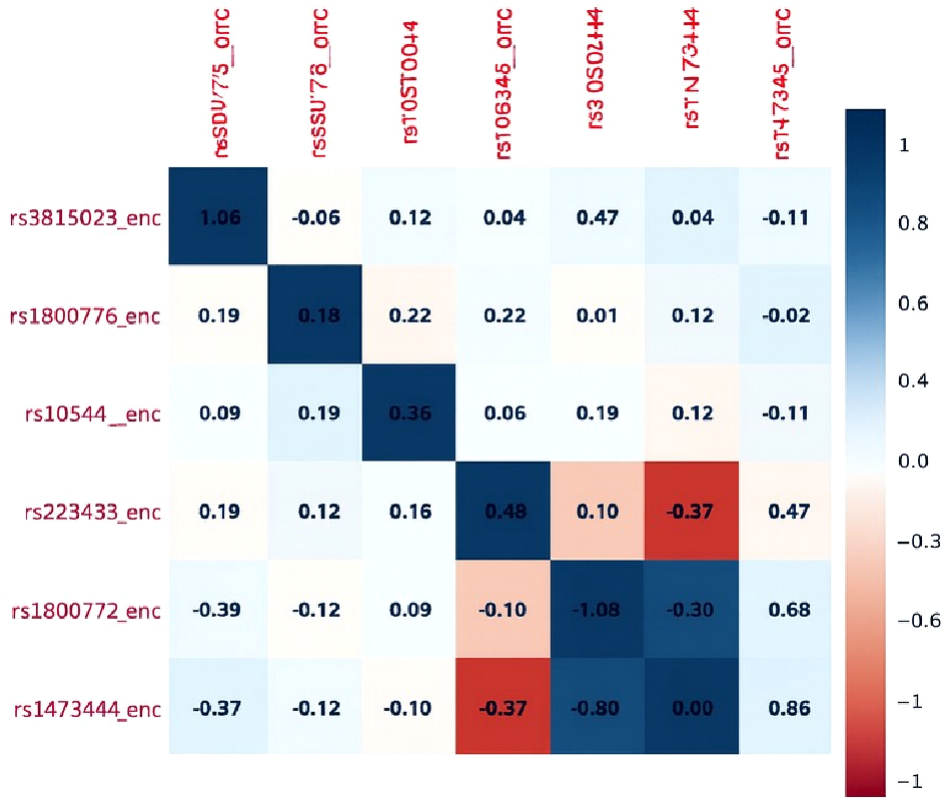
Assessment of Inter-SNP Correlations and Linkage Disequilibrium Patterns. The correlation matrix revealed that most SNPs showed weak or negligible correlations (r values close to 0), indicating a largely independent distribution. Notably, a strong negative correlation was observed between *rs3024490* and *rs1800872* (r = –0.80), suggesting a potential linkage disequilibrium with mutually exclusive inheritance patterns. A few SNP pairs, such as *rs180629* with *rs1474348* (r = 0.19) and *rs1800872* with *rs1800871* (r = 0.26), displayed weak positive correlations. Overall, these findings suggest minimal redundancy among the studied SNPs, supporting their suitability for downstream genetic association analyses.

To evaluate genetic interdependence among the nine target SNPs, a pairwise linkage disequilibrium (LD) matrix was generated (**Figure 2**). Most SNP pairs showed negligible correlation (r² < 0.1), indicating largely independent inheritance within the cohort. However, moderate LD was observed between rs1800872 and rs1800871 (r² ∼ 0.19), suggesting frequent co-inheritance of these IL-10 promoter variants. Additional weaker correlations were noted between rs1800629 and rs1800871 (r² ∼ 0.13), and between rs2430561 and rs1800872 (r² ∼ 0.12). These LD blocks support the co-segregation patterns seen in the genotypic heatmap and justify constructing haplotypes for these non-independent loci to better capture their combined genetic influence.

### 3.6 Polygenic risk score analysis and genetic burden association with disease progression

Statistical analysis did not identify a significant association between the calculated polygenic risk score (PRS) and cervical cancer progression as measured by FIGO stage. The Kruskal-Wallis test showed no statistically significant difference in PRS distributions across the four FIGO stages (χ²(3) = 3.71, p = 0.294). Similarly, Spearman’s rank correlation analysis found no significant monotonic trend between increasing FIGO stage and PRS (ρ = -0.061, p = 0.488). Descriptively, PRS means across stages I to IV were 2.04 (SD = 0.645), 2.23 (SD = 0.907), 1.87 (SD = 0.843), and 2.07 (SD = 0.846), respectively, with the highest median PRS observed in stage II (median = 2.18) and the lowest in stage III (median = 1.76). The overall PRS ranged from 0.29 to 3.98 (mean = 2.055, SD = 0.814) across the entire cohort of 130 patients, while the weighted PRS derived from random-forest feature importance showed high concordance with the unweighted PRS (Spearman ρ = 0.81, p < 0.001). The similar associations seen with the overall survival and patient clustering indicate that the observed prognostic signal is not dependent on equal SNP weighting.

**Figure 3:**
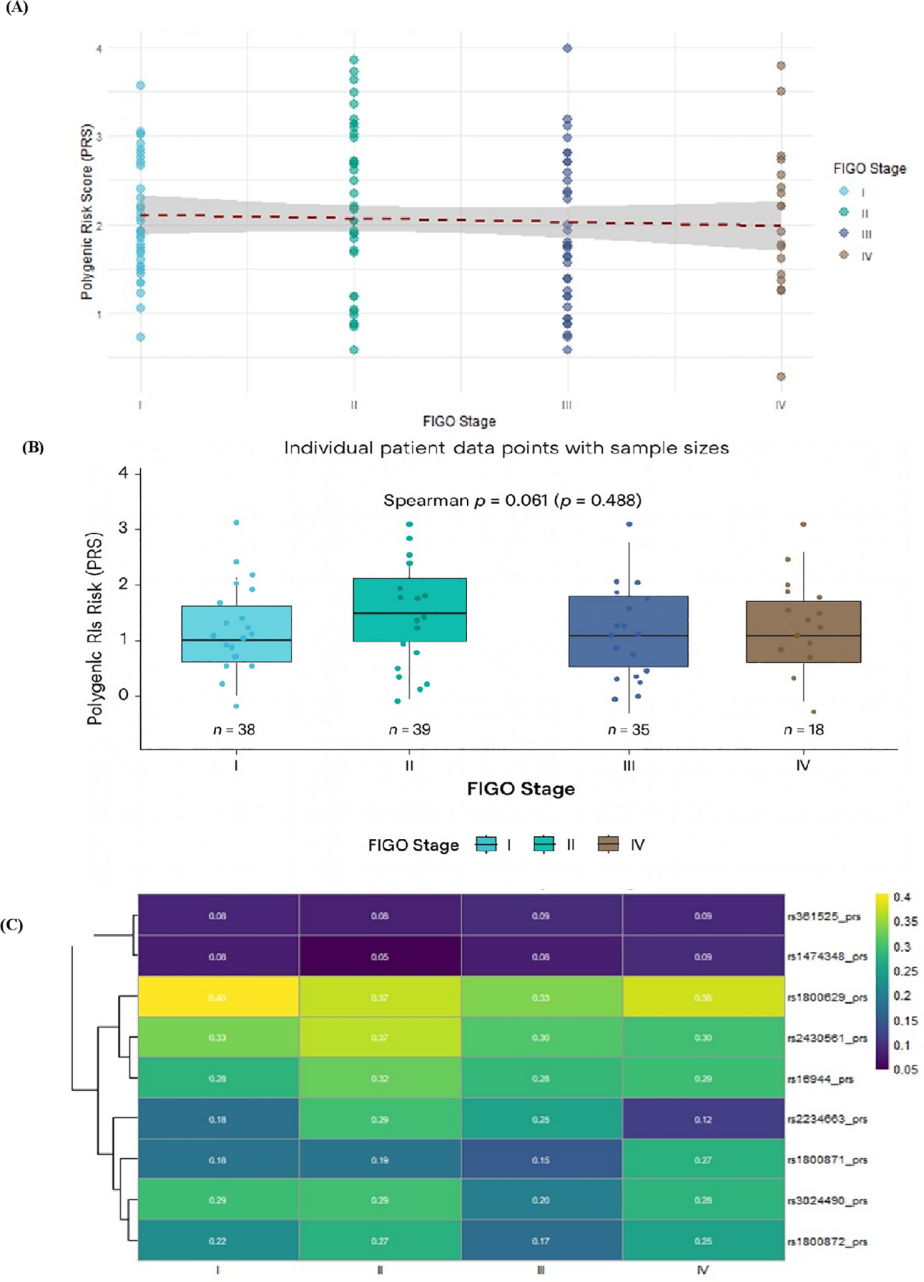
Distribution of Polygenic Risk Scores (PRS) in Cervical Carcinoma Across FIGO Stages. **(A)** Relationship Between Polygenic Risk Score and FIGO Stage (Scatter Plot of PRS vs. FIGO Stage). Individual patient PRS values are plotted for each FIGO stage (I-IV). The solid line represents the linear regression fit, with the shaded area indicating the 95% confidence interval. Dashed horizontal lines mark the median PRS for each stage. Spearman’s rank correlation analysis revealed no significant monotonic trend (ρ = -0.061, p = 0.488). Stage-specific sample sizes: Stage I (n=38), Stage II (n=39), Stage III (n=35), Stage IV (n=18). **(B)** Stratification of PRS Staging in Cervical Canrcinoma (Boxplot Stratification of PRS by FIGO Stage). Boxplots display the median (center line), interquartile range (box), and full range (whiskers) of PRS for each stage, with individual patient data points overlaid. Sample sizes per stage are indicated. The Kruskal-Wallis test indicated no significant difference in PRS distributions across stages (p = 0.294), consistent with the non-significant Spearman correlation (ρ = -0.061, p = 0.488). **(C)** Stage-Specific Mean SNP Contributions to the PRS. Heatmap showing the mean contribution of individual SNPs to the total PRS, stratified by FIGO stage (columns). Each row represents a single SNP. Color intensity reflects the scaled contribution value (see key). Key inflammatory gene variants (e.g., rs1800629 in *TNF-α*, rs2430561 in *IFN-γ*, rs16944 in *IL-1β*) consistently show the highest contributions across all stages. The relative SNP contribution profile remains stable throughout disease progression, with no distinct stage-specific clustering patterns observed.

Visualization of PRS distribution across stages was consistent with the absence of a stage-dependent trend (**Figures 3A, B**). Individual patient data showed substantial overlap in score distributions across FIGO stages I–IV, with no discernible linear progression in the scatter plot (**Figure 3B**); sample sizes: Stage I, n=38; Stage II, n=39; Stage III, n=35; Stage IV, n=18). To explore the underlying genetic architecture, mean SNP-specific contributions to the PRS were analyzed across stages (**Figure 3C**). This heatmap indicated stable relative contributions of individual SNPs regardless of disease severity. Among the variants analyzed, rs1800629 (*TNF-α*), rs2430561 (*IFN-γ*), and rs16944 (*IL-1β*) consistently contributed most strongly to the PRS across all stages. Hierarchical clustering did not reveal any stage-specific genetic signatures.

Collectively, these findings suggest that, in this cohort, the PRS captures a stable genetic profile that does not correlate with clinical stage severity. This leads to the interpretation that the polygenic inflammatory background represented by the PRS may be more relevant to initial disease susceptibility than to subsequent tumor progression, a hypothesis that requires validation in longitudinal or larger cross-sectional cohorts.

### 3.7 Principal component analysis

**Figure 4:**
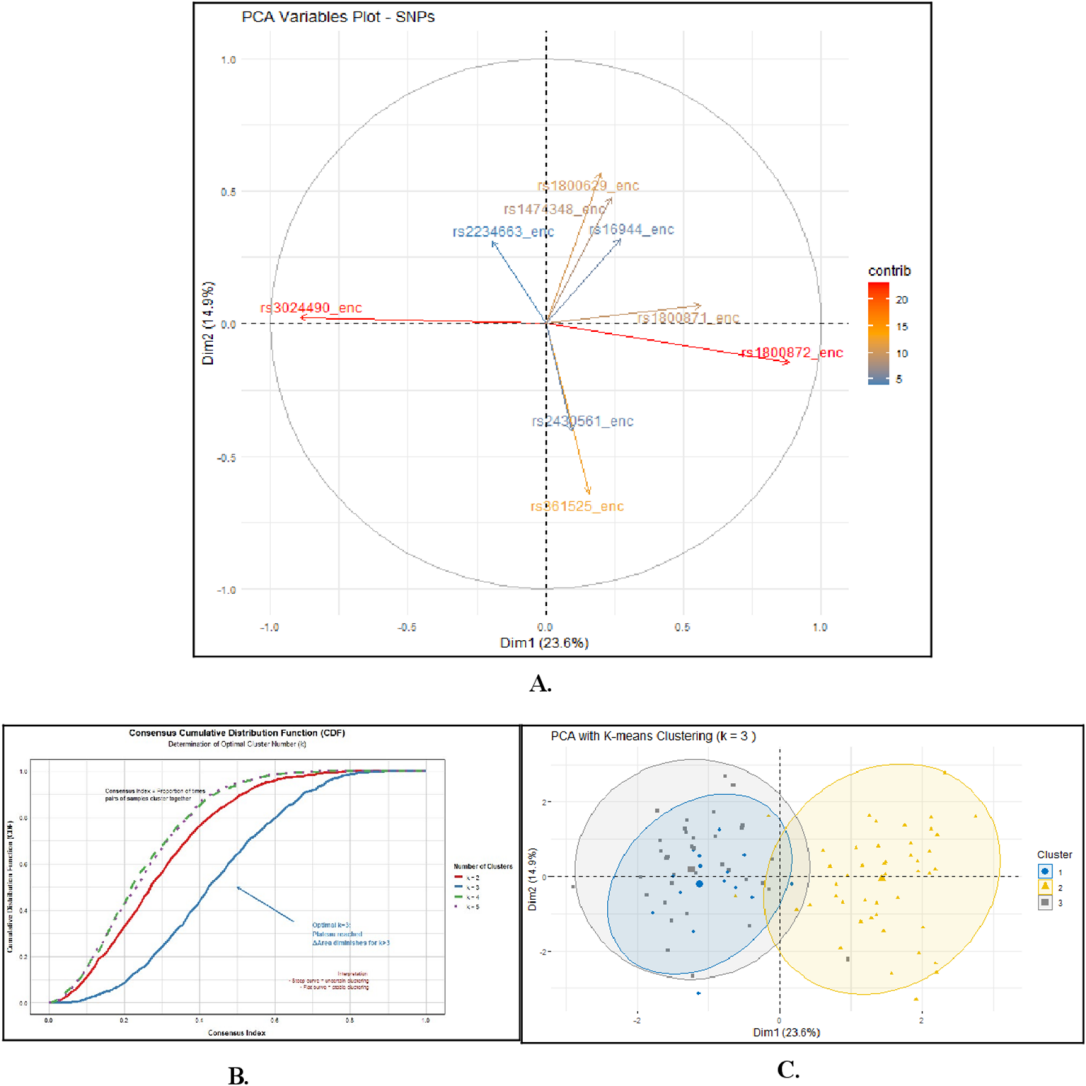
Principal Component Analysis (PCA) of the nine SNPs in Cervical Cancer Cohort. **(A)** PCA of the studied SNPs among CC patients: PCA demonstrates that Dim1 (23.6%) and Dim2 (14.9%) explain 38.5% of total genetic variance. *IL-10* variants rs3024490 and rs1800872 show the strongest yet opposite contributions along Dim1, reflecting their negative correlation and distinct anti-inflammatory effects. *TNF-α* (rs1800629) and *IL-1β* (rs16944) SNPs cluster together, indicating shared pro-inflammatory pathway variability. Vector length indicates contribution strength; color intensity (red = highest, blue = lowest) represents variable importance in defining cervical cancer genetic architecture. **(B)** Consensus Cumulative Distribution Function (CDF) for Determining the Optimal Number of Clusters: Consensus clustering analysis was performed to identify the most stable molecular subgrouping within the cohort. The CDF plot demonstrates that the cumulative distribution curve reached a plateau at *k* = 3, with minimal increase in the area under the curve for higher *k* values. This stability indicates that partitioning the data into three clusters provides the most robust and reproducible classification, which was subsequently applied to the principal component–based genetic dataset. **(C)** PCA with unsupervised K-means clustering (k=3): PCA with K-means clustering partitioned patients into three spatially separated molecular subgroup using Dim1 (23.6%) and Dim2 (14.9%), collectively explaining 38.5% of genetic variance. Cluster 1 (blue circles), Cluster 2 (yellow triangles), and Cluster 3 (gray squares) represent inherent genetic patterns independent of clinical variables. This data-driven classification reveals molecular heterogeneity that potentially correlates with distinct biological behaviors.

To explore the genetic structure within our cohort, we conducted principal component analysis (PCA) on genotypes from the nine candidate SNPs (**Figure 4A****).** The first two principal components explained 38.5% of the total genotypic variance. *IL-10* promoter variants (rs1800871 and rs1800872) loaded strongly onto PC1, while *TNFA* (rs361525) and *IFNG* (rs2430561) variants loaded onto PC2, indicating these sets of variants contributed to distinct axes of variation in this dataset. To identify potential patient subgroups based on these genetic patterns, consensus clustering was performed. The CDF plot (**Figure 4B**) suggested that k = 3 was an optimal partition for this exploratory analysis. Applying k-means clustering (k=3) to the PCA-transformed data revealed three patient clusters with differing SNP allele frequency profiles (**Figure 4C****).** It is important to emphasize that these are data-driven clusters derived from a limited set of candidate SNPs; they represent statistical patterns of genetic variability and should not be equated with biologically validated molecular subgroups. As an exploratory step, these clusters were cross-referenced with available clinical metadata to generate hypotheses about their potential relevance to cervical cancer heterogeneity. Their clinical and biological significance remains to be established through external validation and functional studies.

**Figure 5:**
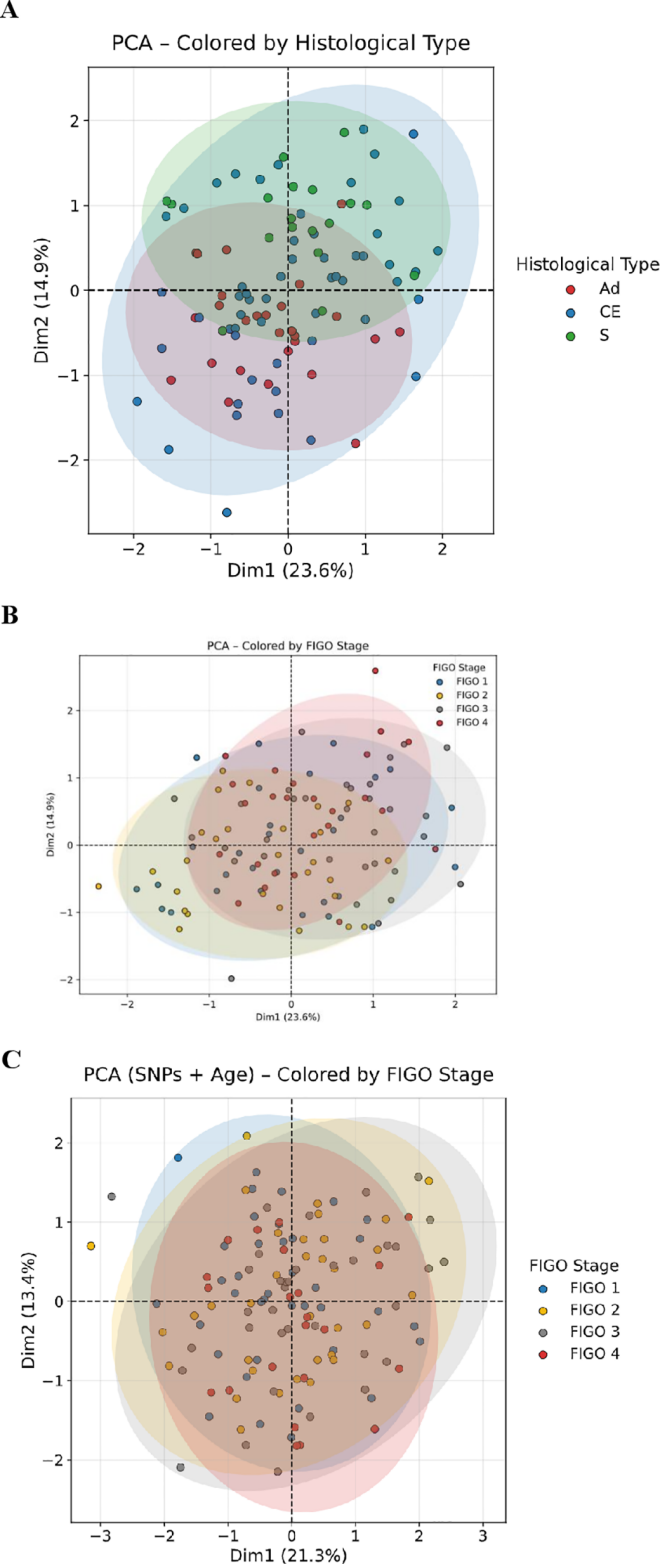
Principal Component Analysis illustrating molecular profile variation across FIGO stages, histological types and age groups. **(A)** Principal Component Analysis of Genotype Data Stratified by FIGO Stage. Scatter plot of the first two principal components derived from genotype data. PC1 and PC2 explain 22.6% and 1.5% of the total variance, respectively. Points are colored by FIGO stage (I–IV). No distinct clustering by stage is observed, with substantial overlap between early-stage (I–II, blue/yellow) and advanced-stage (III–IV, red/grey) cases, indicating that the global genetic variation captured by this SNP set is not strongly associated with disease progression. **(B)** Principal Component Analysis of Genotype Data Stratified by Histological Subtype. The same PCA projection as in panel A, with points colored by the two major histological subtypes: adenocarcinoma (Ad; purple) and squamous cell carcinoma (S; teal). PC1 and PC2 explain 23.0% and 4.0% of the variance, respectively. Both subtypes show broad overlap in the genetic space defined by these components, suggesting no distinct molecular separation between these histological groups based on the selected SNPs. **(C)** Exploratory PCA Incorporating Genotype Data and Patient Age, Stratified by FIGO Stage. Biplot from a PCA performed on genotype data combined with patient age as an exploratory clinical covariate. PC1 and PC2 explain 21.3% and 3.1% of the variance, respectively. Points are colored by FIGO stage (I-IV). Considerable overlap persists across all stages. Although a subtle aggregation of some Stage I samples (blue) is visible, this pattern was not statistically robust, and its biological relevance remains uncertain.

To identify potential multivariate patterns, we performed an exploratory principal component analysis (PCA) integrating clinical and genetic variables (**Figure 5**). When applied to genotype data alone, the first two principal components (PC1 and PC2) accounted for 22.6% and 1.5% of the total variance, respectively (**Figure 5A****).** Coloring the resulting biplot by FIGO stage revealed no distinct clustering; substantial overlap was observed between early-stage (I–II) and advanced-stage (III–IV) cases. This suggests that the global genetic variation captured by the selected SNP set does not strongly correlate with disease stage progression in this cohort.

Similarly, PCA colored by histological subtype adenocarcinoma (Ad) versus squamous cell carcinoma (S) showed no clear separation between subgroups (**Figure 5B****).** Here, PC1 and PC2 explained **23.0%** and **4.0%** of the variance, respectively, with both histological types broadly overlapping in the reduced-dimensional space.

We then conducted an exploratory PCA that incorporated both SNP data and patient age to assess whether adding clinical covariates could improve phenotypic discrimination (**Figure 5C****).** The resulting biplot again exhibited considerable overlap across FIGO stages, with PC1 and PC2 explaining **21.3%** and **3.1%** of the variance. Although minimal aggregation was visible among some early-stage (particularly stage I) samples, this pattern was not statistically robust, and its biological relevance remains unclear.

Collectively, these PCA results indicate that while the selected genetic markers explain moderate variance within the cohort primarily through PC1, they do not provide clear stratification by FIGO stage or histological subtype, either alone or in combination with patient age.

To further examine the PCA results, multidimensional scaling (MDS) was performed using pairwise genetic distances, which revealed a similar spatial organization with limited observable phenotypic separation. Consistent with the PCA, both adenocarcinoma (Ad) and squamous cell carcinoma (S) samples showed considerable overlap in MDS space. Advanced-stage tumors exhibited greater dispersion, suggesting possible increased heterogeneity, but without forming distinct, interpretable clusters.

Taken together, these exploratory multivariate analyses, based on a limited set of candidate SNPs, captured only a modest portion of the total phenotypic variance in this cohort. The consistent absence of clear separation likely reflects the polygenic and heterogeneous nature of cervical cancer, where disease phenotypes are influenced by numerous factors beyond the specific germline variation analyzed here, as well as the inherent limitations of the candidate-gene approach.

**Figure 6:**
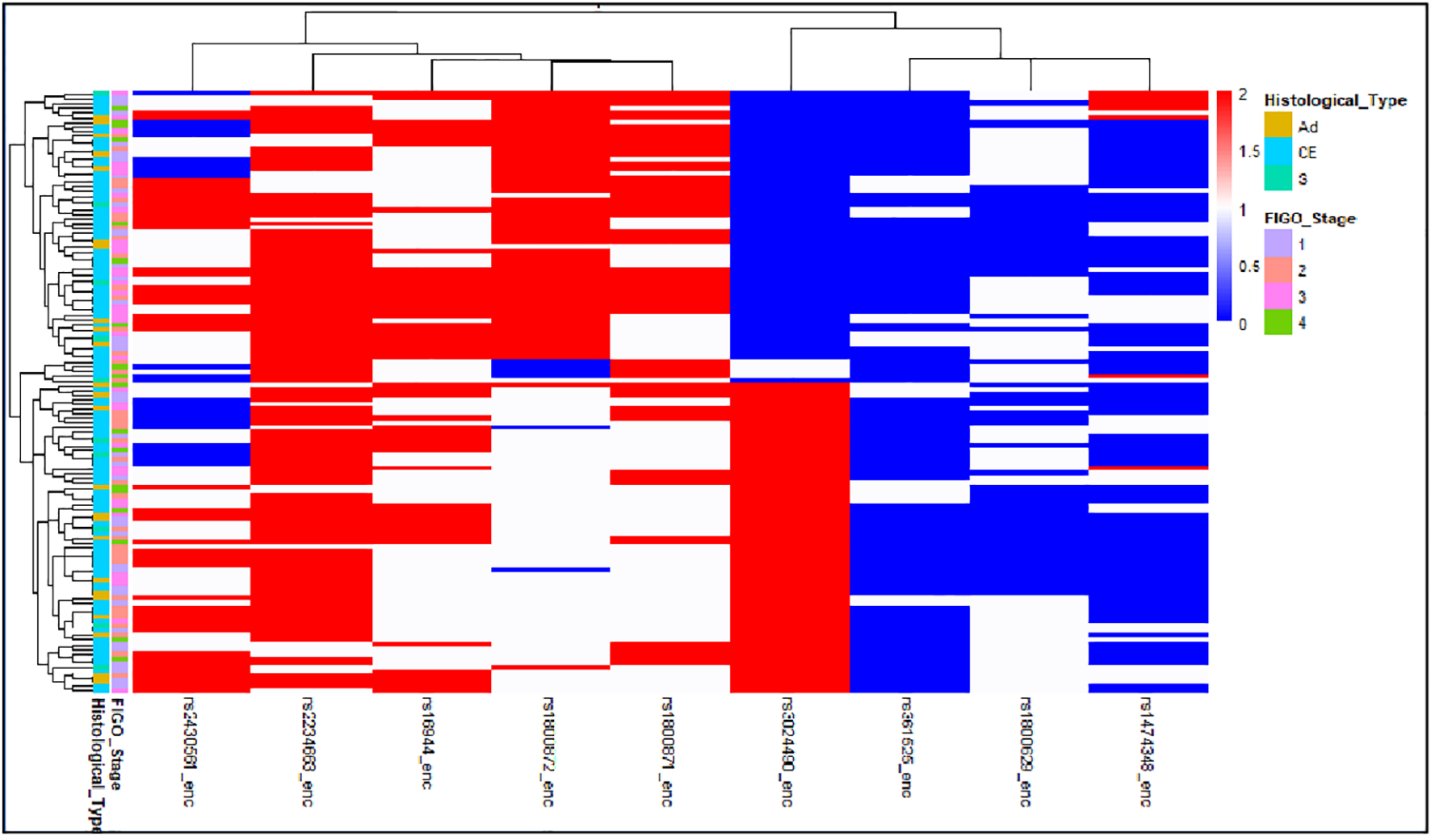
Integrated heatmap of SNP genotypes annotated by clinical and pathological features. Hierarchical clustering displays genotype patterns for nine SNPs across 130 patients (red = homozygous variant, blue = wild-type, white = heterozygous). Annotations indicate histological type (Ad, CE, S) and FIGO stage. Clustering reveals subgroups with shared genetic profiles correlating with histological subgroups, highlighting germline-phenotype interplay.

Finally, a heatmap of SNP genotypes annotated by histology and FIGO stage (**Figure 6**) revealed distinct patterns, especially among adenocarcinoma cases with high prevalence of rs361525 A allele. The heatmap highlights genotype co-occurrence and heterogeneity, supporting the role of germline variation in CC susceptibility.

### 3.8 Machine learning model performance for stage prediction based on inflammatory genetic signature

**Figure 7:**
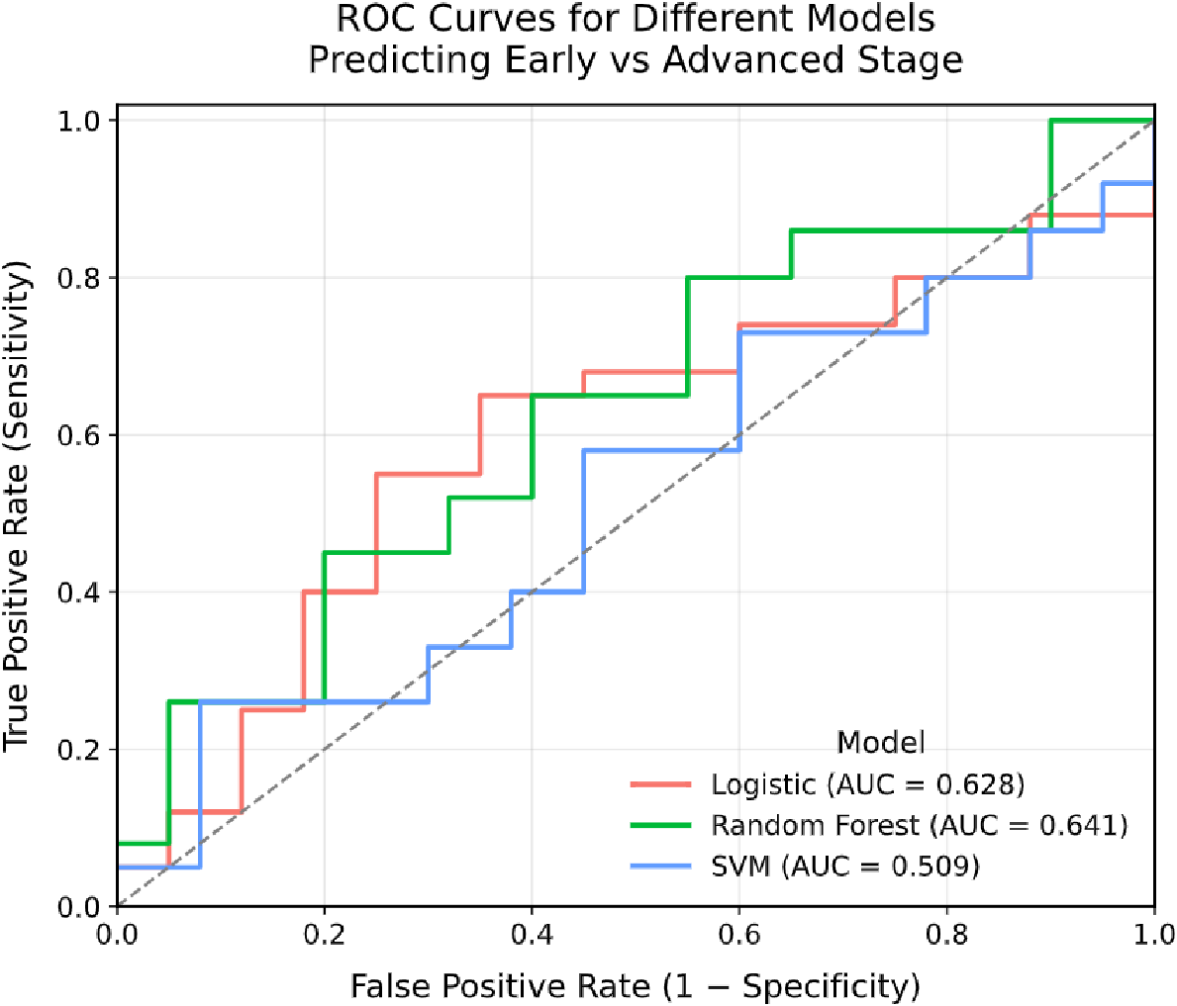
Receiver operating characteristic (ROC) curves for machine learning models predicting early vs. advanced cervical cancer stage based on an inflammatory genetic signature. Curves are shown for logistic regression (LR, AUC = 0.628), random forest (RF, AUC = 0.641), and support vector machine (SVM, AUC = 0.509) classifiers trained on an inflammatory genetic signature. The dashed diagonal line represents the performance of random guessing (AUC = 0.5). The random forest model achieved the highest discriminative ability, though all models showed limited sensitivity in identifying advanced-stage cases.

To explore the potential predictive capacity of the inflammatory gene-derived signature for cervical cancer staging, three machine learning models, Logistic Regression (LR), Random Forest (RF), and Support Vector Machine (SVM), were developed and evaluated in this exploratory analysis. The models were tasked with classifying patients into early (FIGO I–II) versus advanced (FIGO III–IV) stages based on the nine candidate SNPs. The Random Forest model demonstrated the highest discriminative performance among those tested, achieving an area under the receiver operating characteristic curve (AUC) of 0.641, compared to an AUC of 0.628 for Logistic Regression and 0.509 for SVM (**Figure 7**). It should be noted that an AUC of 0.641 represents modest discriminative ability. In terms of classification performance, the Random Forest model attained an accuracy of 68.4% and a high specificity of 95.7%, indicating it was effective in correctly identifying most early-stage cases in this cohort. However, its sensitivity was notably low at 26.7%, suggesting limited utility in identifying true advanced-stage cases. An exploratory threshold optimization analysis of the polygenic risk score (PRS) underlying the signature improved sensitivity to 49.1% while maintaining a specificity of 67.5%.

Overall, these results suggest that, within the constraints of this dataset, the inflammatory genetic signature derived from nine SNPs possesses limited but observable discriminatory power, primarily for distinguishing low-risk patients. The modest performance metrics, particularly the low baseline sensitivity, indicate that this model is not suitable for a standalone clinical application. These findings highlight the exploratory nature of this modelling effort and underscore the need to refine and integrate it with additional clinical or molecular features in future studies to determine whether predictive utility can be improved.

### 3.9 Integrative immunogenetic and pathway modeling of cytokine-mediated disease progression

**Figure 8:**
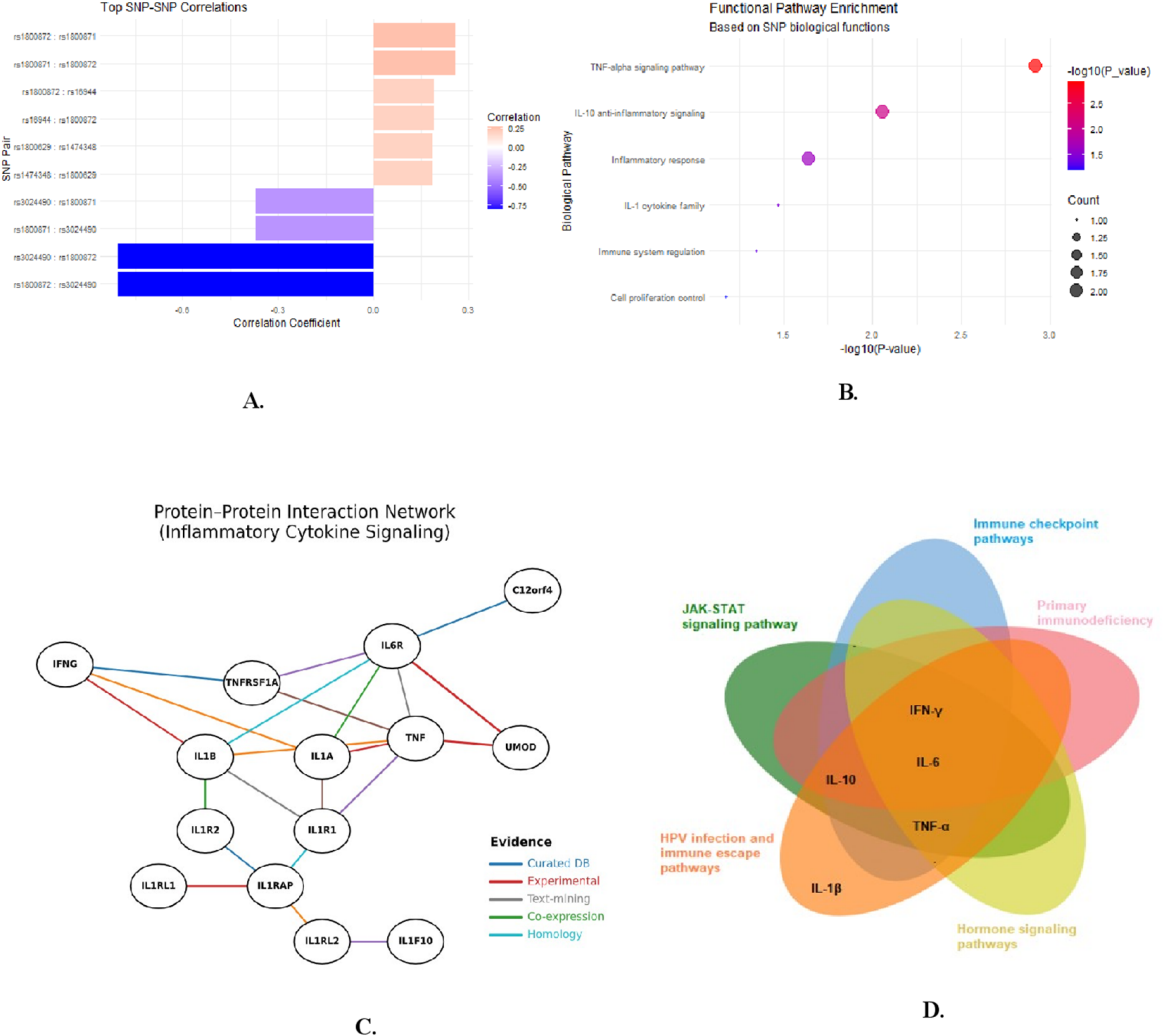
Pathway crosstalk and cytokine network integration. **(A)** Top pairwise correlations between SNPs in cervical cancer patients: Bar plot displays the strongest positive (pink) and negative (blue/purple) correlations among nine SNPs. *IL-10* promoter variants rs1800872 and rs3024490 show a strong negative correlation (r∼0.80), indicating mutually exclusive inheritance. Positive correlations (r∼0.25) include rs1800871-rs1800872, suggesting linkage disequilibrium and co-inheritance patterns within cytokine gene regions. **(B)** Functional pathway enrichment analysis of SNPs associated with cervical cancer: Dot plot displays over-represented biological pathways based on SNP functions. X-axis shows statistical significance (-log10 p-value); dot size indicates gene count; color reflects significance. TNF-α signaling exhibits highest enrichment (p <.01), followed by IL-10 anti-inflammatory and inflammatory response pathways, confirming germline variants functionally concentrate in immune regulation processes underlying cervical cancer pathogenesis. **(C)** Venn diagram illustrating KEGG pathways: The STRING-derived network depicts interactions among major cytokines (TNF-α, IL-1α, IL-1β, IL-6, IL-10, IFN-γ) and their receptors or signaling partners. Highly interconnected nodes, including IL1A, IL1B, IL1R1, and IL1RAP, form the core of an immune–inflammatory module, emphasizing the central role of the IL-1 signaling axis in coordinating downstream immune activation. Strong functional links between TNF–TNFRSF1A and IL6–IL6R highlight cross-talk among proinflammatory and regulatory cytokines, reflecting the balance between immune activation and suppression within the cervical tumor microenvironment. **(D)** Protein–Protein interaction network of inflammatory studied genes: The Venn diagram illustrates the overlap of key cytokines; TNF-α, IL-1β, IL-6, IL-10, and IFN-γ, across major immune and oncogenic signaling pathways, including JAK-STAT signaling, immune checkpoint regulation, HPV infection and immune escape, hormone signaling, and primary immunodeficiency pathways. The central convergence of IL-6 and TNF-α highlights their pivotal roles in linking chronic inflammation with immune evasion, whereas IL-10 and IFN-γ participate in immunoregulatory feedback loops within the tumor microenvironment.

Pairwise SNP correlation analysis revealed several statistically strong positive and negative relationships among cytokine promoter polymorphisms, which could suggest potential genetic interdependencies beyond single-locus effects (**Figure 8A**). The strong linkage between *IL-10* variants rs1800872 and rs1800871 is consistent with their known co-inheritance as a haplotype, while cross-gene correlations such as between rs1800872 (*IL-10*) and rs16944 (*IL-1β*) may reflect population-level genetic architecture rather than direct functional interaction. These observed correlations contribute to the statistical population structure in this cohort and identify variant combinations that warrant further investigation for potential cooperative effects.

Pathway enrichment analysis based on the literature-derived functions of the top-ranked SNPs indicated overrepresentation of immune signaling cascades, primarily involving TNF-α, IL-10, and IL-1 cytokine pathways (**Figure 8B**). The protein-protein interaction (PPI) network analysis of gene products from the studied loci demonstrated a dense immune–inflammatory cluster centered on *IL-1α*, *IL-1β*, *IL-1R1*, and *IL-1RAP*, which theoretically integrates both pro- and anti-inflammatory mediators (**Figure 8C**). The network topology suggests the IL-1 axis and TNF signaling could be influential based on the curated interaction database.

Pathway intersection mapping of the same gene set indicated that the major cytokines (TNF-α, IL-1β, IL-6, IL-10, and IFN-γ) are annotated to multiple overlapping cascades, including JAK–STAT signaling, immune checkpoint regulation, and HPV infection-related pathways (**Figure 8D**). This bioinformatic analysis positions IL-6 and TNF-α as central nodes connecting inflammatory and immune-modulatory processes, while IL-10 and IFN-γ are annotated in regulatory feedback loops. IL-1β was associated with HPV-related and hormone-responsive pathways in the database, consistent with prior literature on its role in chronic inflammation.

Importantly, these pathway and network analyses are based on pre-existing knowledge of the genes harboring the studied SNPs. They do not provide direct functional evidence from this cohort but offer a plausible biological context for interpreting the observed genetic associations, generating hypotheses for future experimental validation.

**Figure 9:**
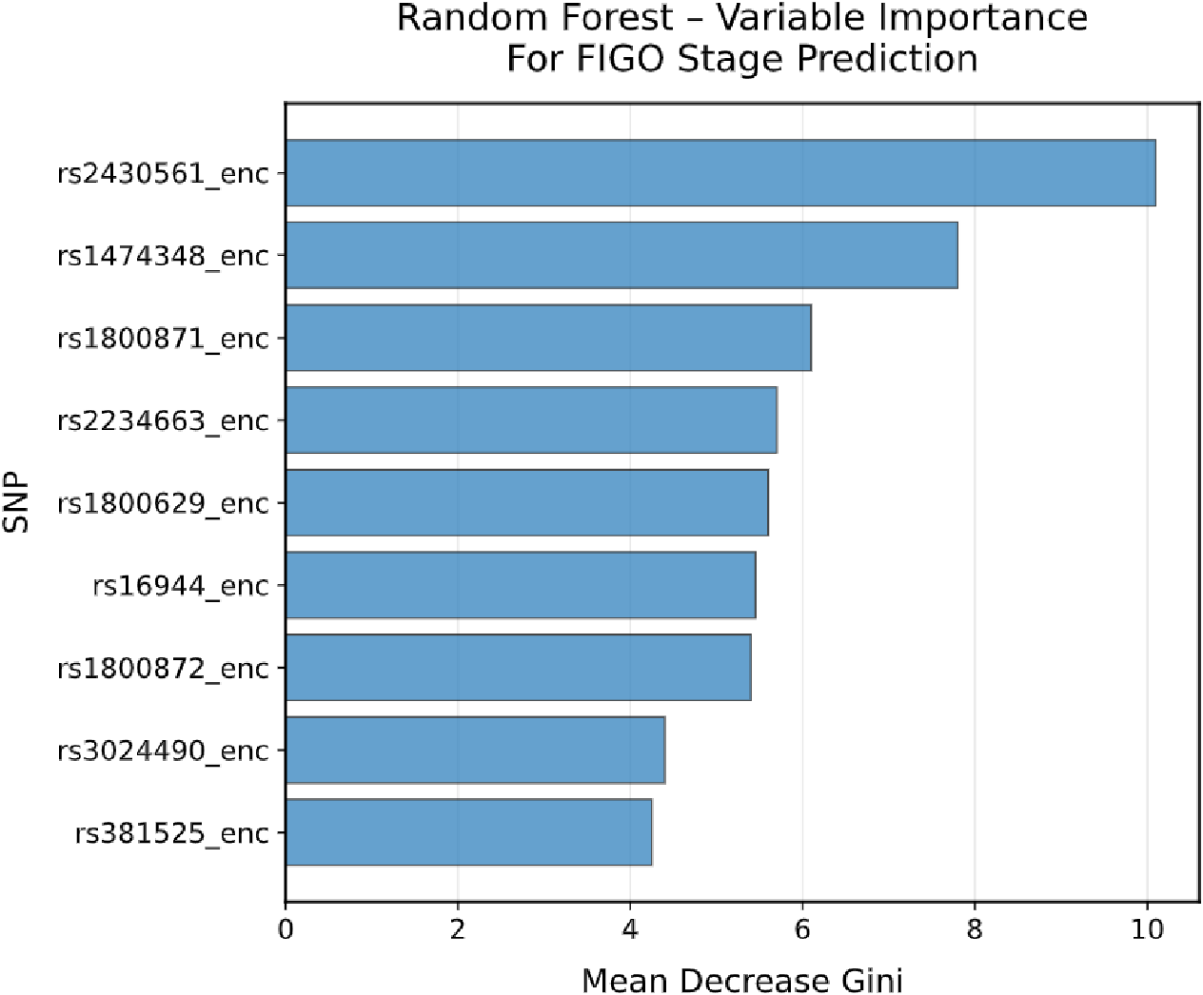
Random Forest variable importance plot for predicting FIGO stage based on immunogenetic SNPs. Bar plot ranks SNPs by Mean Decrease in Gini impurity, indicating contribution to classification accuracy. rs2430561 (*IFN-γ*) shows the highest predictive value, followed by rs1474348 and rs1800871 (*IL-10*). Longer bars indicate greater importance in distinguishing disease stages, revealing germline variants’ predictive power for cervical cancer progression.

To explore potential multigenic effects on disease progression, a random forest model was applied as an exploratory tool to capture non-linear interactions among the nine candidate SNPs. Within the Random Forest model, rs2430561 (*IFN-γ*), rs1474348 (*IL-6*), and rs1800871 (*IL-10*) ranked highest by variable-importance score (**Figure 9**). These rankings indicate that these SNPs contributed most strongly to prediction of FIGO stage in this dataset; however, they should be interpreted solely as statistical features within a classification model rather than evidence of mechanistic primacy. The results are consistent with the hypothesis that immune-related genetic polymorphisms may act in combination to influence clinical phenotype.

Taken together, these integrative bioinformatic analyses—based on a limited set of candidate genes—provide a proposed cytokine-centered network context for the observed genetic associations. This framework suggests a potential connection between inflammation, immune regulation, and viral oncogenesis, generating support for a multigenic immunogenetic model of cervical cancer that remains to be functionally validated.

## 4 Discussion

We present an integrated immunogenetic framework for understanding CC susceptibility and clinical heterogeneity in Tunisian women. Through targeted cytokine genotyping and multivariate machine learning analyses, we demonstrated that host genetic polymorphisms, particularly those in the regulatory regions of *TNF-α, IL-1β*, and *IL-10*, may influence disease risk and outcomes. However, the strength of several associations was modest and requires replication, suggesting that inherited cytokine regulatory variation may be associated with clinical heterogeneity in this cohort, without implying direct effects on immune cell composition or function (47, 48). A distinct immunogenetic profile emerged, with nearly 75% of patients carrying variants in four or more SNPs and 20% exhibiting a high-burden 7-variant signature (rs1800629, rs2430561, rs16944, rs2234663, rs3024490, rs1800872, rs1800871), supporting a polygenic model. The *TNF-α* –238A allele was a borderline risk factor for adenocarcinoma, while *IL-1β* –511T appeared protective, consistent with earlier studies linking cytokine polymorphisms to HPV-related outcomes, although HPV persistence was not measured in this cohort (30, 32).

A derived PRS independently predicted overall survival (49), and integrative analyses defined three germline-based immunogenetic subtypes, establishing immune variation as a key determinant of CC risk and prognosis (41, 45). The PRS was intentionally modeled as an unweighted allelic burden score to reflect cumulative immune-pathway perturbation while minimizing overfitting, a strategy widely used in candidate-gene PRS frameworks. Sensitivity analysis using random-forest–weighted SNPs confirmed that prognostic and clustering results were robust to the weighting strategy. The 7-SNP high-burden signature should be interpreted cautiously, as effect estimates may be inflated due to sample size constraints and multiple comparisons.

Our findings reveal divergent immunogenetic mechanisms underlying cervical cancer histotypes, highlighting the interplay between pro- and anti-inflammatory pathways. The *TNF-α* –238A allele was borderline associated with increased risk of adenocarcinoma but not squamous cell carcinoma, supporting distinct etiological pathways despite shared HPV involvement (30, 50). In contrast, the *IL-1β* –511T allele appeared protective, with complete absence of T/T genotypes among squamous cell carcinoma cases, suggesting subtype-specific protection (51, 52). These opposing effects support a model in which the balance, rather than the magnitude, of inflammatory signaling determines disease trajectory (53, 54). Moreover, interactions between *IL-1RA* VNTR and *IL-10* promoter variants with smoking, menopausal status, and familial cancer history underscore how environmental and hormonal factors modulate genetic susceptibility (52, 55). Collectively, these results indicate that cytokine-mediated inflammation differentially shapes tumor behavior across cervical cancer subtypes through distinct immune microenvironmental dynamics (50, 53, 54).

This study establishes a polygenic risk score (PRS) as an independent prognostic factor for overall survival in cervical cancer (49). Patients in the high-risk tertile (≥7 alleles) exhibited significantly poorer outcomes, independent of FIGO stage, age, and histology, underscoring the prognostic value of cumulative germline immunogenetic variation. Principal component analysis revealed that *IL-10* promoter variants (rs1800871, rs1800872) and *TNF-α*/*IFN-γ* variants define distinct, orthogonal axes of genetic variation, together accounting for 38.5% of total variance. These axes likely reflect divergent immune-regulatory circuits; on one hand dominated by anti-inflammatory IL-10–mediated suppression of cytotoxic responses, and on the other hand by pro-inflammatory TNF-α and IFN-γ signaling promoting chronic inflammation and tissue remodeling (56, 57). Unsupervised K-means clustering identified three immunogenetic subgroups corresponding to unique combinations of these immune pathways, aligning with histological diversity. Complementary random forest analysis pinpointed IFN-γ (rs2430561) and IL-10 (rs1800871, rs1474348) as top predictors of disease stage, highlighting how immune dysregulation shapes both tumor biology and patient outcomes (45, 53, 56).

The integration of machine learning and pathway analyses revealed complementary layers of immune dysregulation in cervical cancer (58). Random forest modeling identified *IFN-γ* rs2430561 and *IL-10* promoter variants as the strongest predictors of FIGO stage within this multivariate framework, highlighting their statistical relevance for classification rather than biological causality (48, 59). The biological plausibility of this finding lies in IFN-γ’s central role in antitumor immunity through regulation of MHC expression, immune cell activation, and tumor growth inhibition. Correlation network analysis further demonstrated strong linkage between *IL-10* promoter variants (rs1800871, rs1800872) and weaker intergenic associations, consistent with coordinated cytokine regulation. Pathway enrichment highlighted convergence on TNF-α, IL-10, and IL-1β signaling, indicating a disturbed equilibrium between pro- and anti-inflammatory axes (43, 54, 56). While these statistical and pathway-based associations are consistent with established immune-regulatory pathways, they do not provide direct evidence of immune microenvironment remodeling in patient tumors (53, 54). Patient clustering into three immunogenetic subgroups underscores that distinct cytokine-driven immune states may underlie histological diversity and therapeutic responsiveness in cervical cancer.

Functional enrichment analyses revealed convergence among TNF-α, IL-10, and IL-1β pathways, underscoring how inherited variants influence the balance between pro-inflammatory and anti-inflammatory responses that govern HPV persistence and malignant progression (56, 60). TNF-α variants may tilt inflammation towards chronic, tumor-promoting states, while IL-10 haplotypes likely hinder immune surveillance, allowing immune evasion (48, 61). Significant gene-environment interactions were also observed, especially between IL-1RA VNTR and tobacco use, menopausal status, and family cancer history, indicating that genetic susceptibility functions within a broader biological context. Whereas the 7-SNP high-risk signature and PRS derived from these findings may be useful for risk stratification pending validation, they should not be viewed as predictive or therapeutic biomarkers at this stage (48). Interpreting the observed gene-environment interactions should be interpreted in the context of previous studies. Cytokine polymorphisms examined, in particular *IL-1RN VNTR*, *TNF-α* promoter variants, and *IL-10* regulatory SNPs, are known to interact with environmental and lifestyle factors relevant to cervical carcinogenesis. *IL-1RN VNTR* alleles are associated with smoking-related inflammatory responses and increased cancer susceptibility (62, 63), while TNF-α −308G>A and −238G>A variants interact with hormonal factors, parity, and tobacco exposure in modulating HPV persistence (64, 65).

In addition, IL-10 rs1800871 and rs1800872 promoter variants influence immune responses linked to HPV infection and cervical neoplasia (66, 67). In contrast, marital status is not biologically linked to cytokine genotypes but serves as an epidemiological proxy for sexual behavior, HPV exposure, and screening access.

Although larger, multiethnic cohorts are needed for replication, these results highlight how germline immune variations and environmental factors jointly influence cervical cancer risk and outcomes. It should be noted that the associations reported here are derived from germline variation and based on statistical modeling. We support the notion that demonstration of immune microenvironment remodeling, effects on HPV persistence, or therapeutic relevance requires integration with tumor transcriptomics, immune-cell profiling, HPV genotyping, and independent cohort validation.

While this study offers an integrative framework combining population genetics, machine learning, and pathway analysis, several limitations warrant mention. The modest sample size (n=130) limits statistical power for subgroup analyses, particularly stratification by histology and FIGO stage, increasing the likelihood of unstable effect estimates. In addition, HPV genotype data were not available for most patients, preventing assessment of virus-host genetic interactions known to influence cervical carcinogenesis. Although false discovery rate control was applied to primary SNP-phenotype comparisons, the combination of limited sample size and multiple stratified analyses means that some nominal associations are likely to represent false positives and should be interpreted cautiously pending replication. The candidate-gene design is limited to capturing only a fraction of the heritable component of immune regulation, making genome-wide approaches preferable, as they provide more complete coverage. The absence of an independent validation cohort limited the generalizability, necessitating functional studies to verify the biological significance of implicated polymorphisms. Although weighted PRS approaches are preferable in large GWAS-derived datasets, effect-size-based weighting is unreliable in small cohorts. Accordingly, we adopted an unweighted PRS as the primary model, supported by sensitivity analyses demonstrating stability of the results.

Despite these limitations, our findings underscore the central role of host immunogenetic factors in CC ontogeny and progression, providing a mechanistic link between genetic predisposition and clinical outcome. Future studies should integrate HPV genotyping, host epigenetic markers, and transcriptomic profiling to refine polygenic models. Collectively, these results advocate for incorporating immunogenetic biomarkers into cervical cancer risk assessment and surveillance programs, particularly in resource-limited settings. The most important limitation of this study is the absence of an independent external validation cohort. Our findings, including the proposed polygenic risk score, require replication in larger, multi-center, and ethnically diverse populations before any clinical relevance can be inferred. The performance metrics reported here are likely optimistic due to overfitting inherent to single-cohort analyses.

## 4 Conclusion

This exploratory study provides preliminary insights into the potential role of germline immune genetic variation in cervical cancer by applying a multi-step analytical framework to clinicopathological and genetic data. By integrating dimensionality reduction, unsupervised clustering, machine learning–based feature selection, and pathway analysis, we moved beyond single-SNP associations to explore how combined host genetic factors may relate to disease heterogeneity.

Our findings suggest that specific promoter polymorphisms in immune-regulatory genes particularly *TNF-α*, *IL-1β*, and *IL-10* may be associated with distinct histological subgroups and disease stages. These variants appear to be involved in key inflammatory and immune-regulatory pathways, hypothetically shaping the tumor microenvironment. Machine learning models highlighted the potential of selected germline variants for distinguishing clinical risk subgroups, supporting their further investigation as candidate biomarkers.

Functional enrichment analyses revealed a potential interconnected cytokine network involving *IL-6–TNF-α* cross-talk, suggesting a model in which coordinated genetic variation in cytokine regulation could influence immune homeostasis and tumor persistence.

Overall, our study proposes a polygenic, immunogenetic framework for understanding cervical cancer susceptibility. The findings, derived from a focused nine-SNP panel in a single cohort, are hypothesis-generating. Future validation in larger, multi-ethnic cohorts, integrated with HPV and somatic genomic data, will be essential to determine the robustness and generalizability of these associations and to assess any eventual clinical relevance.

## Data Availability

The datasets generated and analyzed during the current study are publicly available in the Mendeley repository, accessed via the following DOI: 10.17632/fwftt7ndy5.110.1234/abcd.efghijkl. Additional materials or clarifications can be provided by the corresponding author (Sabrina Zidi) upon reasonable request.
